# Role of MicroRNAs in Human Osteosarcoma: Future Perspectives

**DOI:** 10.3390/biomedicines9050463

**Published:** 2021-04-23

**Authors:** Lola Llobat, Olivia Gourbault

**Affiliations:** 1 Department of Animal Production and Health, Public Veterinary Health and Food Science and Technology, Faculty of Veterinary Medicine, Universidad Cardenal Herrera-CEU, CEU Universities, 46115 Alfara del Patriarca, Spain; 2 Atlantia Veterinary Hospital, 44200 Nantes, France; ogourbault@gmail.com

**Keywords:** biomarkers, human sarcoma, human osteosarcoma, molecular oncology

## Abstract

Osteosarcoma (OS) is a rare form of cancer with high death rate but is one of the most frequent forms of bone cancer in children and adolescents. MiRNAs are small endogenous RNAs that regulate gene expression post-transcriptionally. The discovery of miRNAs could allow us to obtain an earlier diagnosis, predict prognosis and chemoresistance, and lead to the discovery of new treatments in different types of tumors, including OS. Despite the fact that there is currently only one clinical trial being carried out on a single miRNA for solid tumors, it is very probable that the number of clinical trials including miRNAs as prognostic and diagnostic biomarkers, as well as potential therapeutic targets, will increase in the near future. This review summarizes the different miRNAs related to OS and their possible therapeutic application.

## 1. Introduction

Osteosarcoma (OS) is the most common primary bone tumor in children and adolescents. Although advances made in chemotherapy, radiation therapy, and surgery have improved survival rates in different cancers, little to no progress has been seen in recent years for OS [1,2]. Unfortunately, in osteosarcoma, 15–20% of patients admitted for treatment, already have an advanced form of the disease with metastases, that are most commonly found in the lungs [3,4,5]. These lung metastases are what cause the death of the patient in 30–40% of cases [6]. In patients without lung metastases, the 5-year survival rate is 58–75% [7]. This prognosis is determined by the rate of occurrence of metastasis and the appearance of chemoresistance [6]. Given the aggressiveness and high mortality rates of OS, early detection is key to a successful treatment. Recent studies have shown that OS is becoming increasingly resistant to chemotherapeutic agents. Therefore, understanding the underlying molecular mechanisms causing the resistance, and the development of novel and more successful treatments, has become a necessity. In fact, Basile et al. (2020) published a meta-analysis in which they found different prognostic factors in patients under the age of 40, enabling an accurate prediction of the risk a patient has of developing lung metastasis. Optimizing the treatment of patients, based on these different prognostic factors, could play an important role in increasing OS survival rates. [8]. In recent years, microRNAs (miRNAs) have been presented as possible diagnostic and prognostic biomarkers, as modulators of chemoresistance and as a plausible new treatment option in the fight against different tumors, including OS.

## 2. Human Osteosarcoma

Osteosarcoma (OS) is a high-grade malignant bone tumor composed of mesenchymal stem cells (malignant osteoblasts). It is characterized by the deposition of an immature osteoid matrix resulting in the formation of immature woven bone. The incidence of this tumor varies according to race, age, sex, and other factors. It is more frequent in males, children aged 10–14, and in African Americans, suggesting that osteosarcoma may have genetic predisposition [9]. Hereditary disorders such as retinoblastoma, the Li-Fraumeni syndrome, Rothmund–Thomson syndrome, Bloom’s syndrome and Werner syndrome [10], Paget’s disease, chromosomal abnormalities, ionizing radiation, and alkylating agents [11], are risk factors related to the progress of the disease. Current treatment consists of preoperative chemotherapy to shrink the primary tumor and its posterior resection. For the first step, a combination of drugs is commonly used in order to avoid chemoresistance and increase the degree of tumor necrosis. The most common chemotherapeutic drugs include DOX (doxorubicine), cisplatin, ifosfamide, and a high-dose of methotrexate associated with leucovorin calcium as a rescue agent [12]. Three to four weeks after completion of the latter treatment, surgical resection of the tumor is carried out, and 2 weeks after surgery postoperative chemotherapy is administered [13]. Currently, other treatments are in the clinical trial phase, and their results are not yet known. Treatments, currently in phases II and III of clinical trials include, inhibitors of platelet-derived growth factor (PDFG), RANKL and bisphosphonate, vascular endothelial growth factor (VEGF), mTOR, immune checkpoints, antagonists of insulin growth factor 1 receptor (IGF-1R), Tyrosine kinase Src, human epidermal growth factor receptor (HER2/Neu) blockers, cytokines, immunomodulating agents, dendritic cell peptide vaccines, and chimeric antigen receptor (CAR) T cells [14]. Despite advances in treatments, the 5-year survival rate in patients with metastases—which is usually around 50% of patients initiating treatment—has not improved in the last decade and remains below 30% [15,16]. In addition, the number of patients that develop drug resistances are significant. This highlights the importance of finding early diagnostic and prognostic markers and identifying new therapeutic targets in the fight against OS [3].

In the last decade, a new group of molecules known as miRNAs, able to act as genetic biomarkers in numerous diseases including OS, have emerged [17]. They are non-coding RNA molecules of 20–24 base pairs in length, which are unable to code for proteins. They are phylogenetically conserved and have a crucial role in the regulation of gene expression and cellular processes [18,19,20,21,22].

## 3. MicroRNAs: Biogenesis and Biological Functions

### 3.1. Biogenesis of miRNAs

**Figure 1:**
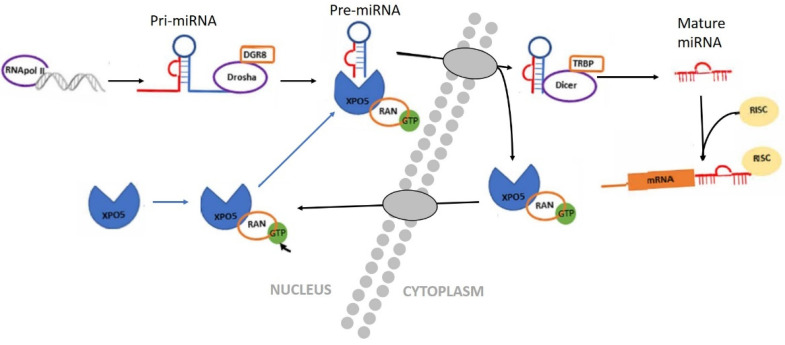
Diagram of miRNA biogenesis. In the nucleus, miRNA that are transcribed by RNA polymerase II or III as primary transcripts (pri-miRNA). The enzyme Drosha and its cofactor DGCR8/Pasha then cut the pri-miRNA molecule to form a pre-miRNA. This pre-miRNA is actively transported from the nucleus to the cytoplasm by the nuclear transport receptor Exportin 5 (XPO5) in a Ran-GTP (guanosine triphosphate) protein dependent manner by XPO5. In the cytoplasm, the pre-miRNA is cut by a second enzyme called Dicer, to form a mature and short double-stranded miRNA molecule. The miRNA duplex unwinds and is incorporated into the RISC protein complex [17].

Biogenesis of miRNAs begins in the cell nucleus, where miRNAs are transcribed by RNA polymerase II or III as primary transcripts (pri-mRNA) to specific genes. Later, the enzyme Drosha and its cofactor DGCR8/Pasha cut this pri-miRNA to form a pre-miRNA (70–90 nucleotides long in the shape of a stem-loop). The nuclear transport receptor Exportin 5 (XPO5), that plays an important role in various cancers, then actively transports the pre-miRNA from the nucleus to the cytoplasm. In the cytoplasm, the pre-miRNA is cut by a Dicer enzyme (regulated by XPO5), to form a mature and short double-stranded miRNA molecule and, after the degradation of one of the two chains, a single strand of miRNA is incorporated into the RISC (RNA-induced silencing complex) protein complex. The RISC protein complex is responsible for directing the silencing of messenger RNA and regulating post transcriptional gene expression, via the inhibition of protein translation or destabilization of target transcripts [23,24,25,26,27,28,29,30,31] (Figure 1).

### 3.2. Biological Functions of miRNAs

MiRNAs are distributed in the intragenic and intergenic regions of the genome. miRNA genes are observed in defined units or clusters [32], containing two or more adjacent sequences with a common transcriptional triggering mechanism. These clusters are involved in different biological functions, as regulators of biological events such as cellular homeostasis, and their aberrant expression has been associated with pathophysiological events [33].

Biological events regulated by miRNAs include differentiation and development, nervous system regulation, immunity, viral infection, DNA repair, cell junctions and cell to cell communication, cellular reprogramming, and metabolism, among others [33]. For example, miR-17/92 is highly expressed in embryonic stem cells and is related to skeletal development, lung morphogenesis, cardiomyocyte and epithelial proliferation, pre-eclampsia and reduction of trophoblast differentiation, and early embryonic lethality [34,35,36,37]. Furthermore, this cluster is associated with B-cell apoptosis, [38] T-cell proliferation [39], DNA repair, post-natal neovascularization [40], mitochondrial metabolism [41], and DNA repair via deacetylation activity of sirtuin 1 (SIRT1) [42]. Other miRNA clusters with different functions include miR-106b/25, related to spermatogonial differentiation [43], generation of new neurons [44], early embryonic lethality [37], coronary artery disease [45], or control of Ca^2+^ accumulation in the mitochondrial matrix [46]. Kabekkodu et al. (2018) published a relevant review of these miRNA clusters among others, and their roles in biological functions [33].

**Figure 2:**
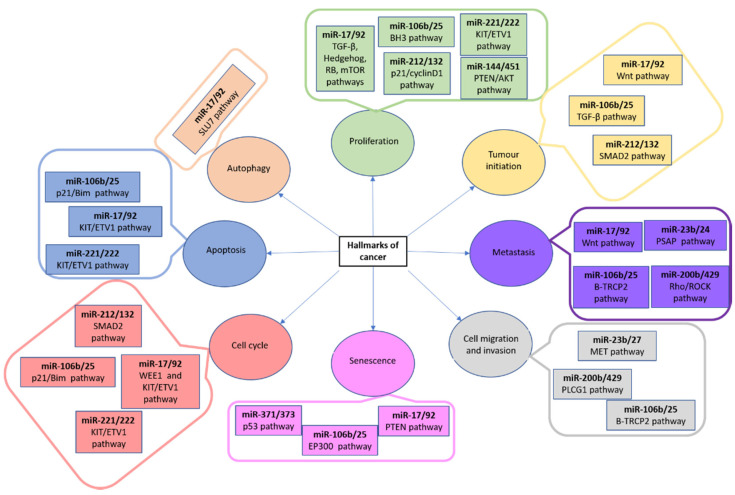
MiRNA clusters, their pathways and associated cancer hallmarks. AKT, AKT serine/threonine kinase; BH3, Bcl-2 homology 3 domain; BIM, Bcl-2-like protein 11; EP300, E1A-asosciated protein p300; ETV1, Ets variant gene 1; KIT, proto-oncogene tyrosine-protein kinase; MET, MET proto-oncogene receptor tyrosine kinase; mTOR, mammalian target of rapamycin complex 1; p21, cyclin dependent kinase inhibitor 1A; PLCG1, phospholipase c gamma 1; PSAP, prosaposin; p53, tumor protein p53; PTEN, phosphatase and tensin homolog; RB1, RB transcriptional corepressor 1; RHO, ROCK Rho-associated protein kinase; SLU7, pre-mRNA splicing factor SLU7; SMAD2, mothers against dpp homolog 2; TRCP2, F-box and WD repeat domain containing 11; TGF, transforming growth factor; WEE1, Wee1A kinase; Wnt, wingless-type mmtv integration site family.

Regarding cancer, the main hallmarks are cell cycle control, apoptosis and senescence, autophagy, cell proliferation and growth, angiogenesis, and metastasis. miRNA clusters play a crucial role in these steps, and their role and type can differ depending on the type of cancer. More specifically, the miR-17/92 and miR106b/25 clusters are related to proliferation, tumor initiation, metastasis, senescence, cell cycle regulation, apoptosis, and autophagy [47,48,49,50,51,52,53,54,55,56,57]. Other miRNA clusters, their pathways, and related cancer hallmarks are shown in Figure 2.

Indeed, some of these clusters are associated with cell cycle progression, migration, and metastasis in osteosarcomas. More specifically, miR-17/92 is found to be abnormally regulated in human OS cell lines and is linked to cell cycle progression [58] and activation of migration and metastasis via the downregulation of the Quaking (QKI) and β-catenin pathways [59]. In fact, patients with a high expression of miR-17/92 presented poor recurrence-free and overall survival rates [60].

### 3.3. MiRNAs and Human Osteosarcoma

Many studies reflect the important role that miRNAs play in the molecular regulation, the appearance and the progression of human OS. Some of these miRNAs act as tumor suppressors, whilst others act as oncogenes [61]. Different miRNAs will act on a diverse range of molecular targets and pathways. Different tumor suppressing miRNAs and their molecular targets in human OS are shown in Table 1.

The presence of onco-miRNAs affecting different targets in human osteosarcoma is demonstrated in Table 2.

Interestingly, miRNAs can act as both tumor suppressors and as onco-miRNAs depending on the target that they act upon. For example, miR-23a can act as tumor suppressor in osteosarcomas, via the inhibition of the special AT-rich-binding protein 1 (SATB1), a gene related to chromatin structure regulation [62]. This finding is supported by Wang et al. who suggests that its role as a tumor suppressor could serve as a potential therapeutic target to fight osteosarcoma. MiR-23a is frequently found to be downregulated in osteosarcoma specimens and cell lines in vitro, which contributes to the aggressiveness of this cancer [63]. Indeed, an experiment carried out by He et al. in an in vivo mice model showed that the downregulation of miR-23a activates the transcription of its target mRNAs, RUNX2 and CXCL12, which play an essential role in OS cellular growth, migration, and invasion [64]. On the contrary, other studies have shown miR-23a acting as an onco-miRNA, and in this case, its downregulation could effectively reduce migration and invasion of osteosarcoma cell lines. As a matter of fact, miR-23a specifically targets the 3′-untranslational regions of PTEN (phosphatase and tensin homolog), lowering its expression. This enhances migration and invasion of osteosarcoma cell lines and might serve as a risk factor for cancer patients [65]. Similar results have been found for miR-20a and miR-107. Depending on which target they bind to, these miRNAs are able to act as both onco-genes or as tumor suppressors. Indeed, the increased expression of miR-20a can reduce colony formation and tumor growth in OS cells, by targeting and suppressing TAK1 expression [66], whereas when this miRNA binds to early growth factor 2 (EGF2) mRNA, it promotes OS cell proliferation and cell cycle [67]. Furthermore, miR-20a seems to participate in the regulation of OS chemoresistance by modulating the activities of the MAPK/ERK and cAMP/PKA signaling pathways [68]. Meanwhile, miR-107 is able to activate MEK/ERK and NF-κB signaling pathways via downregulation of tropomyosin 1 (TPM1). This promotes cell viability, migration, and invasion in human OS cells [69]. Conversely, in vitro studies have demonstrated that miR-107 can inhibit the development of OS via the Wnt/β-catenin signaling pathways, by downregulating Dickkopf-1 (Dkk-1) (Wnt inhibitory factor) [70,71].

## 4. Possible Applications of miRNAs in Human Osteosarcoma

As shown in the previous section, miRNAs have the capacity to act as tumor suppressors and as onco-miRNAs in human osteosarcomas, by acting on different signaling pathways. In fact, more than 177 different miRNAs are found to be differentially expressed in osteosarcoma cell lines [72], and could serve as prognostic, diagnostic, and chemoresistance biomarkers. MiRNAs could also act as possible therapeutic targets to treat human OS. In the following section, the possible uses of certain relevant miRNAs are detailed.

### 4.1. MiRNAs as Potential Biomarkers

One of the most studied miRNAs in osteosarcoma is miR-9. Recently, Wu et al. published a meta-analysis and concluded that a high expression of miR-9 was associated with poor prognosis [73]. Patients with highly expressed levels of miR-9 in osteosarcoma tissues present bigger tumor size, more advanced clinical stages, more frequent distant metastasis, and lower survival rates [74]. The effect that miR-9 has on OS progression seems to be related to it targeting p16 and mediating the activation of the ERK/p38/JNK pathways [75]. Interestingly, the high expression of miR-9, occurs not only in OS tissues, but also in the serum of patients [76]. Similar results have been obtained for the miR-29 family (miR-29a, miR-29b, and miR-29c) [77]. In this case, the underlying mechanism seems to be that miR-29 inhibits the expression of PTEN, thus promoting OS cell proliferation, migration, and metastasis formation [78]. With regards to metastasis occurrence, high circulating levels of miR-21 in patients’ blood sera have been positively correlated with the presence of metastasis and lower 5-year survival rates [79,80]. MiR-95 promotes OS growth by targeting the sodium channel epithelial 1α subunit (SCNN1A) and hepatoma-derived growth factor (HDGF) [81,82]. Other circulating miRNAs found in patients’ sera include miR-95 and miR-194. The higher expression of miR-95 and the lower expression of miR-194 in patients’ sera, contributes to higher metastasis formation and leads to a poorer prognosis in patients with OS [83,84]. These results suggest that altered serum levels of miR-9, miR-21, miR-29, miR-95, and miR-194 have great potential to serve as novel, non-invasive prognostic or diagnostic biomarkers.

Some miRNAs are related to p53, a known oncogene found in many cancers. One of these miRNAs is miR-34a, who’s downregulation in human OS tissues is associated with an unfavorable prognosis [85]. In fact, Xi et al. demonstrated that the downregulation of miR-34 in the tissues of OS patients, inhibited apoptosis through targeting regulated transforming growth factor-β-induced factor homeobox 2s’ (TGIF2) expression [86]. Another miRNA whose expression is lower in OS tissues is miR-126. This miRNA is able to downregulate metalloprotease 9 (ADAM-9), a protease that plays a role in promoting tumorigenesis, cell invasion, and metastasis [87]. Therefore, lower levels of miR-126 could potentially be associated with faster tumor progression, more advanced cancer stages, and shorter overall survival times [88].

MultimiRNA signature for prediction of prognosis of OS patients, has been studied, and three miRNAs (miR-153, miR-212, and miR-591) were used to generate a classified prognosis and prediction signature for OS patients [89]. It was found that miR-153 could potentially act as a tumor suppressor, as high levels of the latter decrease SMAD2, SMAD3, EGFR, and IGFBP-3 expressions, which in turn negatively regulates TGF-β [90]. On the contrary, miR-212 has been seen to inhibit apoptosis and promotes cell proliferation, by upregulating the hedgehog signaling pathway [91]. As for miR-591, although its presence in osteosarcoma has not yet been studied, its function as a tumor suppressor in breast cancer has been suggested, via the inhibition of the Hippo-YAP/TAZ signaling pathway [92]. In addition, miR-591 upregulation has been seen to confer resistance to paclitaxel, a medication used to treat a number of cancers including ovarian cancer. To date however, no research has been carried out on the effects of miR-591 on cisplatin or methotrexate resistance (the most common drugs used to treat OS) [93].

### 4.2. MiRNAs and OS Chemoresistance

One of the most important steps in the human OS is the patient’s response to treatment. The presence of certain miRNAs seems to affect tumor cell chemoresistance. Curiously, high levels of the miR-29 family, which are related to a poor prognosis (see Section 4.1), sensitizes tumor cells against methotrexate and increases the effect of carbon ion radiotherapy [94,95]. The combined use of cisplatin in addition to methotrexate is common when trying to treat osteosarcoma. In a study carried out by Jiang L et al., these two chemotherapy drugs were seen to promote apoptosis in cells that overexpressed miR-126, but not in the silenced osteosarcoma cells [96]. This suggests that the differential expression of miRNAs may play an important role in chemotherapy resistance. Finally, high levels of miR-34a-5p were seen to affect the expression of the tyrosine kinase receptor CD117, thus promoting osteosarcoma’s multidrug resistance via the mTOR pathway [97,98].

### 4.3. MiRNAs as Targets for OS Therapy

Different roles of miR-1 have been studied in diseases such as cardiac diseases, sclerosis, pulmonary arterial hypertension, non-alcoholic fatty liver disease, and cerebral palsy [99,100,101,102]. Furthermore, miR-1 has been related to different types of human cancers such as medulloblastoma, breast cancer, colorectal cancer, and lung cancer [103,104,105,106]. In osteosarcoma, some studies reveal its potential as a therapeutic target; for example, miR-1 is able to suppress growth, proliferation, migration, and invasion of OS cells. In an experiment carried out by Fujii et al., overexpression of miR-1 downregulated PAX3 (and increased p21 levels), and induced arrest in the G_0_/G_1_ phase both in vitro and in vivo [107]. In addition, in vitro studies have shown that miR-1 inhibits the protein expression of vascular endothelial growth factor A (VEGFA), a necessary factor for the vascularization of tumors [108]. These results suggest that miR-1 could be a potential therapeutic target, via the p21 or VEGFA pathways. Regarding the latter pathway, miR-134 (a miRNA of the 14q32 locus) is capable of decreasing VEGFA and VEGFR1 expression in vivo [109], and inhibiting osteosarcoma angiogenesis and proliferation. Moreover, when this microRNA binds to the 3′-UTRs of metalloproteinases 1 and 3 (MMP1 and MMP3), it reduces tumor invasion and metastasis [110]. Therefore, miR-134 could also act as a possible therapeutic target. Other members of the 14q32 locus, such as miR-382, miR-369-3p and miR-544, suppress the expression of the c-MYC (oncogene) in human OS cells [111]. Studies in patient OS cells have demonstrated that another MMP, more precisely MMP16, is upregulated by miR-145 [112]. However, MMP16 is not miR-154′s only target. Other molecules, such as ROCK1, VEGF, and HDAC4 are also targets of this miRNA [113,114,115]. High levels of miR-145 are seen to inhibit cell proliferation, invasion, and metastasis in OS. A recent meta-analysis carried out by Xu et al. [116] showed that the level at which miR-145 was expressed in different tumors was correlated with patient prognosis, however this was not shown in patients with OS. Nevertheless, other studies related the downregulation of miR-145 with advanced tumor progression and poor prognosis in OS [117]. Another microRNA, miR-21, has been put forward as alternative possible therapeutic target. Indeed, miR-21 suppresses PTEN expression, and induces apoptosis in human osteosarcoma cells [118].

There are a large number of studies providing relevant information about the possible functions of microRNAs as potential biomarkers for prognosis, diagnosis and resistance to chemotherapy or as possible therapeutic targets in different cancers. Despite the good potential OS has, as a target of future clinical trials based on miRNA therapy, the truth is that there are actually only very few clinical trials based on miRNAs in this cancer to date. Possible explanations for this are that as a rare form cancer, there are only very few patients and therefore the recruitment potential for clinical trials is lower than it is in other cancer types. In addition, obtaining miRNAs from a bone microenvironment requires complicated procedures. Finally, the complexity of the human genome makes it difficult to interpret the results obtained from miRNA trials. We are still uncertain of the effects each miRNA may exert on the rest of the genome, and resistance mechanisms are likely. As a matter of fact, some clinical trials for solid tumors based on miRNA have been carried out. One of them was based on the therapeutic molecule MRX34, whose target is miR-34a. The results of MRX34 treatment with dexamethasone premedication did show some clinical activity without serious toxicity. However, some patients presented severe cytokine release syndrome, and the trial was terminated [119]. The final results of this clinical trial are not published yet. Another clinical trial, based on miR-16 (a tumor suppressor miRNA), has been carried out in lung cancer and mesothelioma, and included 27 patients. Although the safety of the trial was acceptable, twenty of patients died during the clinical trial due to tumor progression [120]. The results found in these different clinical trials indicate the amount of knowledge that still needs to be acquired on miRNAs before they can be used as biomarkers or as cancer therapies, including data on their safety and effectiveness.

Currently, there are a total of 334 clinical trials addressing miRNAs as possible biomarkers or therapeutic targets in different types of cancer. However, only one of them is for osteosarcoma (NCT01190943). This clinical trial is studying DNA biomarkers, including miRNAs, in tissue samples from patients with osteosarcoma. Other clinical trials, based on different miRNAs in the hope of treating further pathologies are currently being carried out. More specifically, currently, two clinical trials with therapeutic molecules targeting miRNAs in other pathologies are being carried out, and they are showing promising results. These molecules target miR-122 for the treatment of the hepatitis C viral infection and are in phases 1 and 2 of clinical trials [121,122].

## 5. Conclusions

OSs are a rare form of cancer with high death rates in humans, mainly children. The discovery of miRNAs marked a new era in molecular biology and their presence can give valuable information about the physiopathology of osteosarcoma. The relationship between different miRNAs and patient prognosis, cancer diagnosis, cancer progression, chemotherapy resistance, and their use as possible new drug targets have been demonstrated. However, there is currently only one clinical trial related to the inhibition of miRNA in solid tumors; this miRNA is miR-34a. It seems that one of the issues is the delivery system of the therapies. Another issue is the localized delivery of miRNA therapeutics. The hope is that in the near future these drawbacks will be overcome, and anti-cancer therapy based on miRNAs will be a reality, especially for solid tumors such as OS, with poor prognosis and high mortality rates.

## Figures and Tables

**Table 1:** Different tumor suppressing miRNAs downregulated and their targets in human osteosarcoma (OS) (data recovered by [61]).

Target	miRNAs
Aurora-B	Let-7
ESR1	miR-1
Bcl-2	miR-15a, miR-34a, miR-125b, miR-143, miR-190b, miR-326, miR-449a
IGF system	miR-16, miR-26a, miR-133a, miR-150, miR-497
MED27	miR-18a
TAK1	miR-20a
VEGF	miR20b, miR-29b, miR-205, miR-410
HMG system	miR-22, miR-106a-5p, miR-142-3p, miR-505
SATB1	miR-23a, miR-376a-3p
LPAATβ	miR-24
SOX4, SOX9 or SOX2	miR-25, miR-25-3p, miR-132, miR-188, miR-212, miR-32 (SOX9), miR-336 (SOX2)
PFKFB3	miR-26
PTEN	miR-30a
HIF1	miR-33b, miR-20b, miR186
MARCKS	miR-34c-3p
EZRIN	miR-96, miR-144, miR-150
TNFAIP	miR-15a, miR-99a,
PI3K-AKT	miR-100, miR-497
mTOR	miR-101
Β-catenin	miR-107, miR-184
TGF β	miR-124, miR-153, miR-422a
TGF-α	miR-205, miR-376a, miR-376c, miR-422a,
ANXA2	miR-206
ROR2	miR-208b
ROCK1	miR-129-5p, miR-139, miR-144, miR-145, miR-148a, miR-150, miR-198, miR-214-5p
CDK14	miR-216a
CDK6	miR-377, miR-494, miR-3928, miR-494
PDK1	miR-379
LRH-1	miR-381, miR-451
ELF2	miR-409-3p
ZEB1	miR-126, miR-130a, miR-141, miR-200b, miR-429, miR-643
SETD8	miR-127-3p
Sirt1	miR-133b, miR-138, miR-204
c-Myc	miR-135b, miR-449c
MTDH	miR-136
FXYD6	miR-137
HDAC4	miR-140
GLI2	miR-141-3p, miR-202
FASN	miR-142-3p
FOSL2	miR-143-3p
E2F1	miR-320
E2F3	miR-152, miR-874
Wnt system	miR-154, miR-184, miR-217
TIAM1	miR-182
LRP6	miR-183
ZERB2	miR-187
TCF7	miR-192
CDH2	miR-194
NKD1	miR-195-5p
VANGL-2	miR-199-3p
PMP22	miR-200bc/429
RAB22A	miR-203
ANXA2	miR-206
ROR2	miR-208b
Hsp90B1	miR-223
Ect-2	miR-223
Rac-1	miR-224
ADAM9	miR-302a
LDHA	miR-323a-3p
Rab10	miR-329
Bmi-1 (or BMI1)	miR-330-3p, miR-452
Survivin	miR-335
AEG-1	miR-342-3p, miR-448, miR-506
KRAS	miR-365
FOXM1	miR-370
FOXP4	miR-491-5p
SATB1	miR-376a-3p
LRH-1	miR-451
c-Met	miR-454, miR-613
PKC	miR-486
HMGA2	miR-490-3p
HMGB1	miR-505
HMGN5	miR-495
TSPAN1	miR-491-3p
PAK6	miR-492
ARL2	miR-497-5p
FGF2	miR-503
AKT-1 (or Akt)	miR-520a-3p, miR-564
MMP8	miR-539
ITGAV	miR-548c-3p
KLF5	miR-590-5p
PDGFB	miR-598
YAP-1	miR-625
PIM1	miR-638
CYC1	miR-661
RAB23	miR-665
BCL9	miR-1301
ERBB3	miR-3928

Abbreviations: ESR1 (Estrogen Receptor 1), Bcl-2 (B Cell Lymphoma), IGF (Insulin Growth Factor), MED27 (Mediator Complex subunit 27), TAK1 (Mitogen-activated protein 3 kinase 7), VEGF (Vascular Endothelial Growth Factor), HMG (High Mobility Group), SATB1 (Special AT-rich sequence-binding protein 1), LPAATβ (Lysophosphatidic acid acyltransferase β), SOX (Sex Determining region Y-box), PFKFB3 (6-phosphofructo-2-kinase/fructuose-2,6-biphosphatase-3), PTEN (Phosphatase and Tensin homolog), HIF1 (Hypoxia-inducible transcription factor-1), MARCKS (Myristoylated alanine-rich protein kinase C substrate), TNFAIP (Tumor Necrosis Factor-α-induced protein), PI3K-AKT (Phosphatidil Inositol 3-Kinase), TGF (Transforming Growth Factor), ANXA2 (Annexin 2), ROR2 (Receptor Tyrosine kinase-like Orphan Receptor 2), ROCK1 (Rho associated Coiled-Coil containing protein Kinase 1), CDK (Cyclin-dependent kinase), PDK (Pyruvate Dehydrogenase Kinase), LRH-1 (Liver Receptor Homolog-1), ELF2 (E74-Like Factor 2), ZEB1 (Zinc Finger E-box Homeobox 1), Sirt1 (Sirtuin 1), MTDH (Metadherin), FXYD6 (FXYD Domain containing Ion Transport Regulator 6), HDAC4 (Histone Deacetylase 4), FASN (Fatty Acid Synthase), FOSL2 (FOS-Like antigen 2), TIAM1 (T-cell Lymphoma Invasion and Metastasis-inducing protein 1), LRP6 (Lipoprotein Receptor-related Protein 6), ZERB2 (Zinc finger E-box Binding Homeobox 2), TCF7 (T cell-specific transcription factor 7), CDH2 (Cadherin-2), NKD1 (Naked Cuticle Homolog 1), VANGL-2 (Van Gogh-Like 2), PMP22 (Peripheral Myelin Protein 22), RAB22A (Ras-related protein Rab-22A), Hsp90 (Heat Shock protein 90), Ect-2 (Epithelial Cell Transforming 2), LDHA (Lactate Dehydrogenase A), AEG-1 (Astrocyte-Elevated Gene 1), KRAS (Kirsten Rat Sarcoma viral oncogene), FOX (Forkhead Box Protein), PKC (Protein Kinase C), TSPAN1 (Tetraspanin 1), PAK6 (p-21 Activated Kinase 6), ARL2 (ADP Ribosylation factor-Like protein 2), FGF2 (Fibroblast Growth Factor 2), MMP (Matrix Metalloproteinase), ITGAV (Integrin Alpha V), KLF5 (Kruppel-Like Factor 5), PDGFB (Plateled Derived Growth Factor B), YAP-1 (Yes-associated protein 1), PIM1 (Moloney murine Leukemia virus 1), CYC1 (Cyclin 1), RAB23 (Ras-related protein Rab-23), BCL9 (B-cell CLL/lymphoma 9), ERBB3 (erb-b2 receptor tyrosine kinase 3).

**Table 2:** Different onco-miRNAs upregulated and their targets in human OS (data recovered by [61]).

Target	miRNAs
GCIP	miR-9
KLF4	miR-10b
PTEN	miR-17, miR-21, miR-23a, miR-92a, miR-93, miR-196a, miR-214, miR-221, miR-1908
BRCC2	miR-17-5p, miR-603
SOCS6	miR-19
EGR2	miR-20a
ING5	miR-27-3p
TET1	miR-27a-3p
TGF-β	miR-29
RASSF1A	miR-81a
RECK	miR-92b
CDKN1	miR95-3p
VNN2	miR-106a
PI3K	miR-106b
TPM1	miR-107
PPARγ	miR-130b
CDC14A	miR-131a
ROCK1	miR-138
BMP9	miR-149
HBP1	miR-155
RIPK1	miR-155-5p
CFIm25	miR-181a
Chk2	miR-191
p21	miR-93, miR-95-3p
p27	miR-199a-5p
BRD7	miR-300
DAB2IP	miR-367
FOXO1 and FOXO4	miR-374a, miR-660, miR-664 (FOXO4)
KLF9	miR-378
MTSS1	miR-411
Bim	miR-488
TP53INP1	miR-504
VANGL-2	miR-542-3p, miR-542-5p
MAP3K9	miR-1247
AGTR1	miR-1248
SRSF3	miR-1908-5p

Abbreviations: GCIP (Glutamate receptor-interacting protein), KLF4 (Kruppel-Like Factor 4), PTEN (Phosphatase and Tensin homolog), BRCC2 (Breast Cancer Cell 2), SOCS6 (Suppressor of Cytokine Signaling 6), EGR2 (Early Growth Response 2), ING5 (Inhibitor of Growth Family member 5), TET1 (Ten Eleven Translocation 1), TGF-β (Transforming Growth Factor β), RASSF1A (Ras Association domain Family 1 isoform A), RECK (Reversion inducing Cysteine Rich protein with Kazal motifs), CDKN1 (Cyclin Dependent Kinase inhibitor 1), VNN2 (Vanine 2), PI3K (Phosphatidil Inositol 3 Kinase), TPM1 (Tropomyosin 1), PPARγ (Peroxisome Proliferator-Activated Receptor γ), CDC14A (Dual specificity protein phosphatase 14A), ROCK1 (Rho associated Coiled-Coil containing protein Kinase 1), BMP9 (Bone Morphogenetic Protein 9), HBP1 (HMG-Box Transcription Factor 1), RIPK1 (Receptor-Interacting serine/threonine-Protein Kinase 1), CFIm25 (Component of the Cleavage Factor Im25), Chk2 (Checkpoint Kinase 2), BRD7 (Bromodomain Containing 7), DAB2IP (Disable homolog 2-Interacting Protein), FOXO (Forkhead box Protein O), KLF9 (Krueppel-Like Factor 9), MTSS1 (MTSS I-BAR Domain Containing 1), Bim (Bcl-2-Like Protein 11), TP53INP1 (Tumor Protein p53-Inducible Nuclear Protein 1), VANGL-2 (VANGL Planar Cell Polarity Protein 2), MAP3K9 (Mitogen-Activated Protein Kinase Kinase Kinase 9), AGTR1 (Angiotensin II Receptor Type 1), SRSF3 (Serine and Arginine Rich Splicing Factor 3).

## Data Availability

Not applicable.
